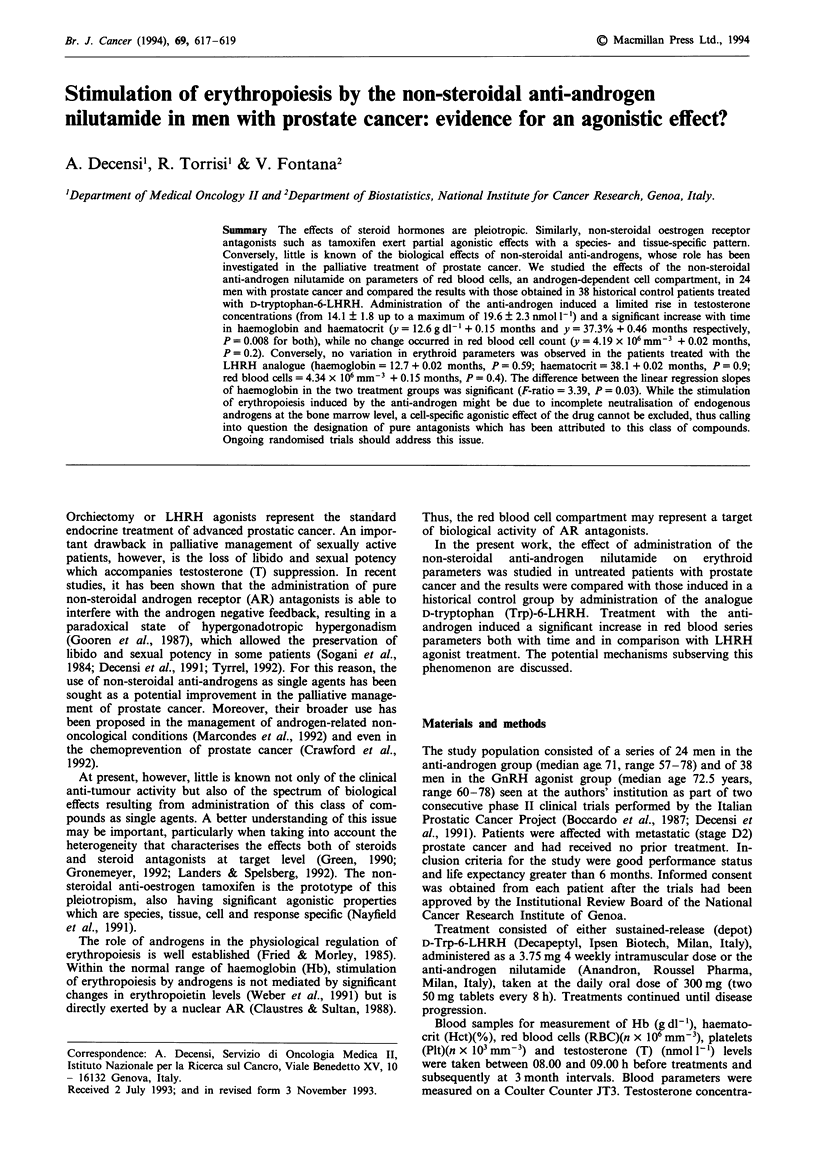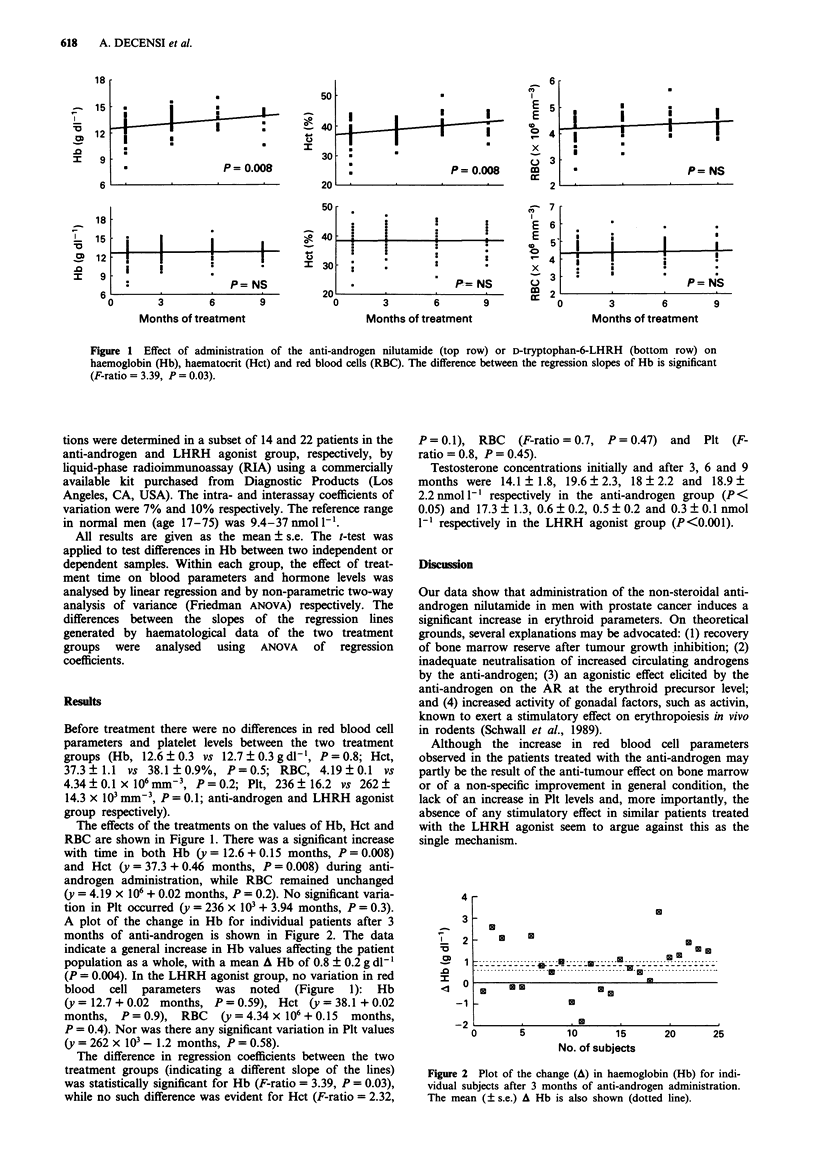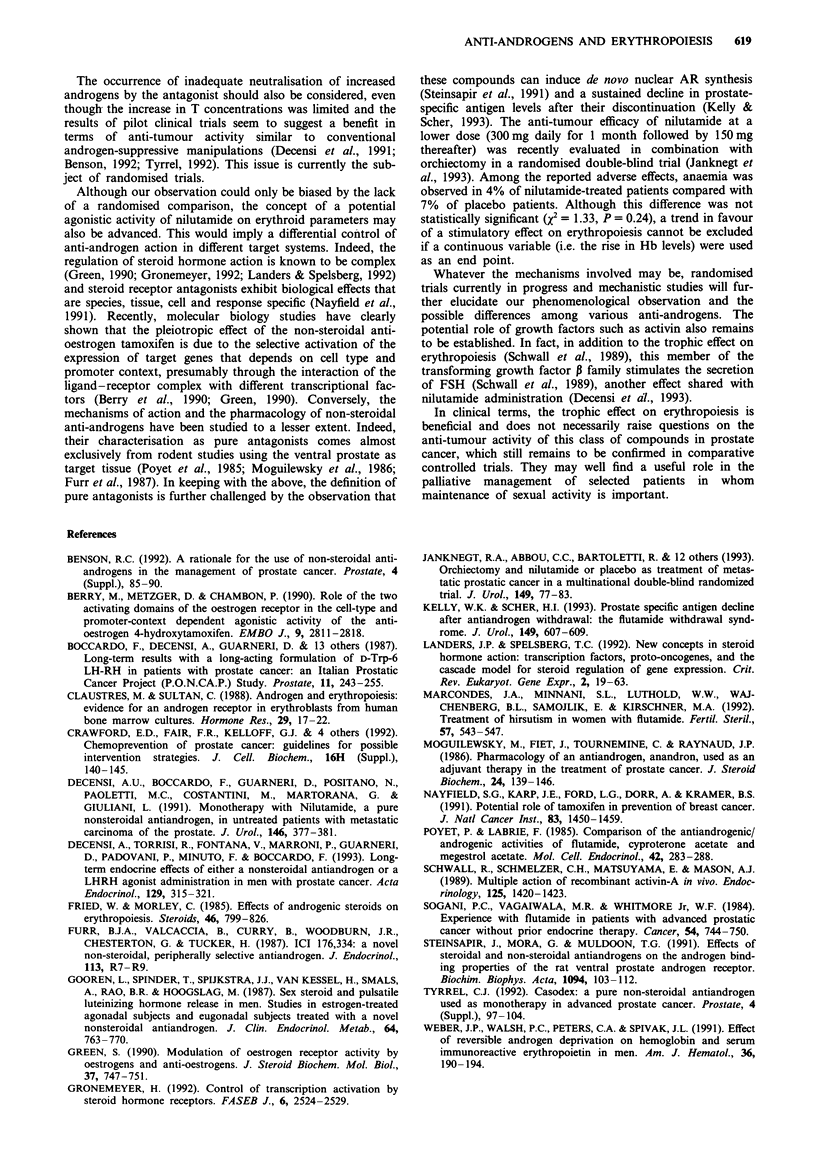# Stimulation of erythropoiesis by the non-steroidal anti-androgen nilutamide in men with prostate cancer: evidence for an agonistic effect?

**DOI:** 10.1038/bjc.1994.115

**Published:** 1994-03

**Authors:** A. Decensi, R. Torrisi, V. Fontana

**Affiliations:** Department of Medical Oncology II, National Institute for Cancer Research, Genoa, Italy.

## Abstract

The effects of steroid hormones are pleiotropic. Similarly, non-steroidal oestrogen receptor antagonists such as tamoxifen exert partial agonistic effects with a species- and tissue-specific pattern. Conversely, little is known of the biological effects of non-steroidal anti-androgens, whose role has been investigated in the palliative treatment of prostate cancer. We studied the effects of the non-steroidal anti-androgen nilutamide on parameters of red blood cells, an androgen-dependent cell compartment, in 24 men with prostate cancer and compared the results with those obtained in 38 historical control patients treated with D-tryptophan-6-LHRH. Administration of the anti-androgen induced a limited rise in testosterone concentrations (from 14.1 +/- 1.8 up to a maximum of 19.6 +/- 2.3 nmol l-1) and a significant increase with time in haemoglobin and haematocrit (y = 12.6 g dl-1 + 0.15 months and y = 37.3% + 0.46 months respectively, P = 0.008 for both), while no change occurred in red blood cell count (y = 4.19 x 10(6) mm-3 + 0.02 months, P = 0.2). Conversely, no variation in erythroid parameters was observed in the patients treated with the LHRH analogue (haemoglobin = 12.7 + 0.02 months, P = 0.59; haematocrit = 38.1 + 0.02 months, P = 0.9; red blood cells = 4.34 x 10(6) mm-3 + 0.15 months, P = 0.4). The difference between the linear regression slopes of haemoglobin in the two treatment groups was significant (F-ratio = 3.39, P = 0.03). While the stimulation of erythropoiesis induced by the anti-androgen might be due to incomplete neutralisation of endogenous androgens at the bone marrow level, a cell-specific agonistic effect of the drug cannot be excluded, thus calling into question the designation of pure antagonists which has been attributed to this class of compounds. Ongoing randomised trials should address this issue.


Br. J. Cancer (1994), 69, 617-619 © Macmillan Press Ltd., 1994

### Stimulation of erythropoiesis by the non-steroidal anti-androgen nilutamide in men with prostate cancer: evidence for an agonistic effect?

A. Decensi[1], R. Torrisi[1] & V. Fontana[2]

[1]Department of Medical Oncology II and [2]Department of Biostatistics, National Institute for Cancer Research, Genoa, Italy.

**Summary** The effects of steroid hormones are pleiotropic. Similarly, non-steroidal oestrogen receptor antagonists such as tamoxifen exert partial agonistic effects with a species- and tissue-specific pattern. Conversely, little is known of the biological effects of non-steroidal anti-androgens, whose role has been investigated in the palliative treatment of prostate cancer. We studied the effects of the non-steroidal anti-androgen nilutamide on parameters of red blood cells, an androgen-dependent cell compartment, in 24 men with prostate cancer and compared the results with those obtained in 38 historical control patients treated with D-tryptophan-6-LHRH. Administration of the anti-androgen induced a limited rise in testosterone concentrations (from $14.1 \pm 1.8$ up to a maximum of $19.6 \pm 2.3$ nmol $l^{-1}$) and a significant increase with time in haemoglobin and haematocrit ($y = 12.6$ g $dl^{-1}$ + 0.15 months and $y = 37.3\%$ + 0.46 months respectively, $P = 0.008$ for both), while no change occurred in red blood cell count ($y = 4.19 \times 10^6$ $mm^{-3}$ + 0.02 months, $P = 0.2$). Conversely, no variation in erythroid parameters was observed in the patients treated with the LHRH analogue (haemoglobin = 12.7 + 0.02 months, $P = 0.59$; haematocrit = 38.1 + 0.02 months, $P = 0.9$; red blood cells = $4.34 \times 10^6$ $mm^{-3}$ + 0.15 months, $P = 0.4$). The difference between the linear regression slopes of haemoglobin in the two treatment groups was significant (*F*-ratio = 3.39, $P = 0.03$). While the stimulation of erythropoiesis induced by the anti-androgen might be due to incomplete neutralisation of endogenous androgens at the bone marrow level, a cell-specific agonistic effect of the drug cannot be excluded, thus calling into question the designation of pure antagonists which has been attributed to this class of compounds. Ongoing randomised trials should address this issue.

Orchiectomy or LHRH agonists represent the standard endocrine treatment of advanced prostatic cancer. An important drawback in palliative management of sexually active patients, however, is the loss of libido and sexual potency which accompanies testosterone (T) suppression. In recent studies, it has been shown that the administration of pure non-steroidal androgen receptor (AR) antagonists is able to interfere with the androgen negative feedback, resulting in a paradoxical state of hypergonadotropic hypergonadism (Gooren *et al.*, 1987), which allowed the preservation of libido and sexual potency in some patients (Sogani *et al.*, 1984; Decensi *et al.*, 1991; Tyrrel, 1992). For this reason, the use of non-steroidal anti-androgens as single agents has been sought as a potential improvement in the palliative management of prostate cancer. Moreover, their broader use has been proposed in the management of androgen-related non-oncological conditions (Marcondes *et al.*, 1992) and even in the chemoprevention of prostate cancer (Crawford *et al.*, 1992).

At present, however, little is known not only of the clinical anti-tumour activity but also of the spectrum of biological effects resulting from administration of this class of compounds as single agents. A better understanding of this issue may be important, particularly when taking into account the heterogeneity that characterises the effects both of steroids and steroid antagonists at target level (Green, 1990; Gronemeyer, 1992; Landers & Spelsberg, 1992). The non-steroidal anti-oestrogen tamoxifen is the prototype of this pleiotropism, also having significant agonistic properties which are species, tissue, cell and response specific (Nayfield *et al.*, 1991).

The role of androgens in the physiological regulation of erythropoiesis is well established (Fried & Morley, 1985). Within the normal range of haemoglobin (Hb), stimulation of erythropoiesis by androgens is not mediated by significant changes in erythropoietin levels (Weber *et al.*, 1991) but is directly exerted by a nuclear AR (Claustres & Sultan, 1988). Thus, the red blood cell compartment may represent a target of biological activity of AR antagonists.

In the present work, the effect of administration of the non-steroidal anti-androgen nilutamide on erythroid parameters was studied in untreated patients with prostate cancer and the results were compared with those induced in a historical control group by administration of the analogue D-tryptophan (Trp)-6-LHRH. Treatment with the anti-androgen induced a significant increase in red blood series parameters both with time and in comparison with LHRH agonist treatment. The potential mechanisms subserving this phenomenon are discussed.

### Materials and methods

The study population consisted of a series of 24 men in the anti-androgen group (median age 71, range 57-78) and of 38 men in the GnRH agonist group (median age 72.5 years, range 60-78) seen at the authors' institution as part of two consecutive phase II clinical trials performed by the Italian Prostatic Cancer Project (Boccardo *et al.*, 1987; Decensi *et al.*, 1991). Patients were affected with metastatic (stage D2) prostate cancer and had received no prior treatment. Inclusion criteria for the study were good performance status and life expectancy greater than 6 months. Informed consent was obtained from each patient after the trials had been approved by the Institutional Review Board of the National Cancer Research Institute of Genoa.

Treatment consisted of either sustained-release (depot) D-Trp-6-LHRH (Decapeptyl, Ipsen Biotech, Milan, Italy), administered as a 3.75 mg 4 weekly intramuscular dose or the anti-androgen nilutamide (Anandron, Roussel Pharma, Milan, Italy), taken at the daily oral dose of 300 mg (two 50 mg tablets every 8 h). Treatments continued until disease progression.

Blood samples for measurement of Hb (g $dl^{-1}$), haematocrit (Hct)(%), red blood cells (RBC)($n \times 10^6$ $mm^{-3}$), platelets (Plt)($n \times 10^3$ $mm^{-3}$) and testosterone (T) (nmol $l^{-1}$) levels were taken between 08.00 and 09.00 h before treatments and subsequently at 3 month intervals. Blood parameters were measured on a Coulter Counter JT3. Testosterone concentra-

Correspondence: A. Decensi, Servizio di Oncologia Medica II, Istituto Nazionale per la Ricerca sul Cancro, Viale Benedetto XV, 10 - 16132 Genova, Italy.
Received 2 July 1993; and in revised form 3 November 1993.

**Figure 1** Effect of administration of the anti-androgen nilutamide (top row) or D-tryptophan-6-LHRH (bottom row) on haemoglobin (Hb), haematocrit (Hct) and red blood cells (RBC). The difference between the regression slopes of Hb is significant ($F$-ratio = 3.39, $P$ = 0.03).

tions were determined in a subset of 14 and 22 patients in the anti-androgen and LHRH agonist group, respectively, by liquid-phase radioimmunoassay (RIA) using a commercially available kit purchased from Diagnostic Products (Los Angeles, CA, USA). The intra- and interassay coefficients of variation were 7% and 10% respectively. The reference range in normal men (age 17–75) was 9.4–37 nmol $l^{-1}$.

All results are given as the mean ± s.e. The $t$-test was applied to test differences in Hb between two independent or dependent samples. Within each group, the effect of treatment time on blood parameters and hormone levels was analysed by linear regression and by non-parametric two-way analysis of variance (Friedman ANOVA) respectively. The differences between the slopes of the regression lines generated by haematological data of the two treatment groups were analysed using ANOVA of regression coefficients.

### Results

Before treatment there were no differences in red blood cell parameters and platelet levels between the two treatment groups (Hb, 12.6 ± 0.3 *vs* 12.7 ± 0.3 g dl$^{-1}$, $P$ = 0.8; Hct, 37.3 ± 1.1 *vs* 38.1 ± 0.9%, $P$ = 0.5; RBC, 4.19 ± 0.1 *vs* 4.34 ± 0.1 × $10^6$ mm$^{-3}$, $P$ = 0.2; Plt, 236 ± 16.2 *vs* 262 ± 14.3 × $10^3$ mm$^{-3}$, $P$ = 0.1; anti-androgen and LHRH agonist group respectively).

The effects of the treatments on the values of Hb, Hct and RBC are shown in Figure 1. There was a significant increase with time in both Hb ($y$ = 12.6 + 0.15 months, $P$ = 0.008) and Hct ($y$ = 37.3 + 0.46 months, $P$ = 0.008) during anti-androgen administration, while RBC remained unchanged ($y$ = 4.19 × $10^6$ + 0.02 months, $P$ = 0.2). No significant variation in Plt occurred ($y$ = 236 × $10^3$ + 3.94 months, $P$ = 0.3). A plot of the change in Hb for individual patients after 3 months of anti-androgen is shown in Figure 2. The data indicate a general increase in Hb values affecting the patient population as a whole, with a mean Δ Hb of 0.8 ± 0.2 g dl$^{-1}$ ($P$ = 0.004). In the LHRH agonist group, no variation in red blood cell parameters was noted (Figure 1): Hb ($y$ = 12.7 + 0.02 months, $P$ = 0.59), Hct ($y$ = 38.1 + 0.02 months, $P$ = 0.9), RBC ($y$ = 4.34 × $10^6$ + 0.15 months, $P$ = 0.4). Nor was there any significant variation in Plt values ($y$ = 262 × $10^3$ − 1.2 months, $P$ = 0.58).

The difference in regression coefficients between the two treatment groups (indicating a different slope of the lines) was statistically significant for Hb ($F$-ratio = 3.39, $P$ = 0.03), while no such difference was evident for Hct ($F$-ratio = 2.32, $P$ = 0.1), RBC ($F$-ratio = 0.7, $P$ = 0.47) and Plt ($F$-ratio = 0.8, $P$ = 0.45).

Testosterone concentrations initially and after 3, 6 and 9 months were 14.1 ± 1.8, 19.6 ± 2.3, 18 ± 2.2 and 18.9 ± 2.2 nmol $l^{-1}$ respectively in the anti-androgen group ($P$ < 0.05) and 17.3 ± 1.3, 0.6 ± 0.2, 0.5 ± 0.2 and 0.3 ± 0.1 nmol $l^{-1}$ respectively in the LHRH agonist group ($P$ < 0.001).

### Discussion

Our data show that administration of the non-steroidal anti-androgen nilutamide in men with prostate cancer induces a significant increase in erythroid parameters. On theoretical grounds, several explanations may be advocated: (1) recovery of bone marrow reserve after tumour growth inhibition; (2) inadequate neutralisation of increased circulating androgens by the anti-androgen; (3) an agonistic effect elicited by the anti-androgen on the AR at the erythroid precursor level; and (4) increased activity of gonadal factors, such as activin, known to exert a stimulatory effect on erythropoiesis *in vivo* in rodents (Schwall *et al.*, 1989).

Although the increase in red blood cell parameters observed in the patients treated with the anti-androgen may partly be the result of the anti-tumour effect on bone marrow or of a non-specific improvement in general condition, the lack of an increase in Plt levels and, more importantly, the absence of any stimulatory effect in similar patients treated with the LHRH agonist seem to argue against this as the single mechanism.

**Figure 2** Plot of the change (Δ) in haemoglobin (Hb) for individual subjects after 3 months of anti-androgen administration. The mean (± s.e.) Δ Hb is also shown (dotted line).

The occurrence of inadequate neutralisation of increased androgens by the antagonist should also be considered, even though the increase in T concentrations was limited and the results of pilot clinical trials seem to suggest a benefit in terms of anti-tumour activity similar to conventional androgen-suppressive manipulations (Decensi *et al.*, 1991; Benson, 1992; Tyrrel, 1992). This issue is currently the subject of randomised trials.

Although our observation could only be biased by the lack of a randomised comparison, the concept of a potential agonistic activity of nilutamide on erythroid parameters may also be advanced. This would imply a differential control of anti-androgen action in different target systems. Indeed, the regulation of steroid hormone action is known to be complex (Green, 1990; Gronemeyer, 1992; Landers & Spelsberg, 1992) and steroid receptor antagonists exhibit biological effects that are species, tissue, cell and response specific (Nayfield *et al.*, 1991). Recently, molecular biology studies have clearly shown that the pleiotropic effect of the non-steroidal anti-oestrogen tamoxifen is due to the selective activation of the expression of target genes that depends on cell type and promoter context, presumably through the interaction of the ligand–receptor complex with different transcriptional factors (Berry *et al.*, 1990; Green, 1990). Conversely, the mechanisms of action and the pharmacology of non-steroidal anti-androgens have been studied to a lesser extent. Indeed, their characterisation as pure antagonists comes almost exclusively from rodent studies using the ventral prostate as target tissue (Poyet *et al.*, 1985; Moguilewsky *et al.*, 1986; Furr *et al.*, 1987). In keeping with the above, the definition of pure antagonists is further challenged by the observation that these compounds can induce *de novo* nuclear AR synthesis (Steinsapir *et al.*, 1991) and a sustained decline in prostate-specific antigen levels after their discontinuation (Kelly & Scher, 1993). The anti-tumour efficacy of nilutamide at a lower dose (300 mg daily for 1 month followed by 150 mg thereafter) was recently evaluated in combination with orchiectomy in a randomised double-blind trial (Janknegt *et al.*, 1993). Among the reported adverse effects, anaemia was observed in 4% of nilutamide-treated patients compared with 7% of placebo patients. Although this difference was not statistically significant ($\chi^2 = 1.33$, $P = 0.24$), a trend in favour of a stimulatory effect on erythropoiesis cannot be excluded if a continuous variable (i.e. the rise in Hb levels) were used as an end point.

Whatever the mechanisms involved may be, randomised trials currently in progress and mechanistic studies will further elucidate our phenomenological observation and the possible differences among various anti-androgens. The potential role of growth factors such as activin also remains to be established. In fact, in addition to the trophic effect on erythropoiesis (Schwall *et al.*, 1989), this member of the transforming growth factor $\beta$ family stimulates the secretion of FSH (Schwall *et al.*, 1989), another effect shared with nilutamide administration (Decensi *et al.*, 1993).

In clinical terms, the trophic effect on erythropoiesis is beneficial and does not necessarily raise questions on the anti-tumour activity of this class of compounds in prostate cancer, which still remains to be confirmed in comparative controlled trials. They may well find a useful role in the palliative management of selected patients in whom maintenance of sexual activity is important.

### References

BENSON, R.C. (1992). A rationale for the use of non-steroidal anti-androgens in the management of prostate cancer. *Prostate*, **4** (Suppl.), 85–90.

BERRY, M., METZGER, D. & CHAMBON, P. (1990). Role of the two activating domains of the oestrogen receptor in the cell-type and promoter-context dependent agonistic activity of the anti-oestrogen 4-hydroxytamoxifen. *EMBO J.*, **9**, 2811–2818.

BOCCARDO, F., DECENSI, A., GUARNERI, D. & 13 others (1987). Long-term results with a long-acting formulation of D-Trp-6 LH-RH in patients with prostate cancer: an Italian Prostatic Cancer Project (P.O.N.CA.P.) Study. *Prostate*, **11**, 243–255.

CLAUSTRES, M. & SULTAN, C. (1988). Androgen and erythropoiesis: evidence for an androgen receptor in erythroblasts from human bone marrow cultures. *Hormone Res.*, **29**, 17–22.

CRAWFORD, E.D., FAIR, F.R., KELLOFF, G.J. & 4 others (1992). Chemoprevention of prostate cancer: guidelines for possible intervention strategies. *J. Cell. Biochem.*, **16H** (Suppl.), 140–145.

DECENSI, A.U., BOCCARDO, F., GUARNERI, D., POSITANO, N., PAOLETTI, M.C., COSTANTINI, M., MARTORANA, G. & GIULIANI, L. (1991). Monotherapy with Nilutamide, a pure nonsteroidal antiandrogen, in untreated patients with metastatic carcinoma of the prostate. *J. Urol.*, **146**, 377–381.

DECENSI, A., TORRISI, R., FONTANA, V., MARRONI, P., GUARNERI, D., PADOVANI, P., MINUTO, F. & BOCCARDO, F. (1993). Long-term endocrine effects of either a nonsteroidal antiandrogen or a LHRH agonist administration in men with prostate cancer. *Acta Endocrinol.*, **129**, 315–321.

FRIED, W. & MORLEY, C. (1985). Effects of androgenic steroids on erythropoiesis. *Steroids*, **46**, 799–826.

FURR, B.J.A., VALCACCIA, B., CURRY, B., WOODBURN, J.R., CHESTERTON, G. & TUCKER, H. (1987). ICI 176,334: a novel non-steroidal, peripherally selective antiandrogen. *J. Endocrinol.*, **113**, R7–R9.

GOOREN, L., SPINDER, T., SPIJKSTRA, J.J., VAN KESSEL, H., SMALS, A., RAO, B.R. & HOOGSLAG, M. (1987). Sex steroid and pulsatile luteinizing hormone release in men. Studies in estrogen-treated agonadal subjects and eugonadal subjects treated with a novel nonsteroidal antiandrogen. *J. Clin. Endocrinol. Metab.*, **64**, 763–770.

GREEN, S. (1990). Modulation of oestrogen receptor activity by oestrogens and anti-oestrogens. *J. Steroid Biochem. Mol. Biol.*, **37**, 747–751.

GRONEMEYER, H. (1992). Control of transcription activation by steroid hormone receptors. *FASEB J.*, **6**, 2524–2529.

JANKNEGT, R.A., ABBOU, C.C., BARTOLETTI, R. & 12 others (1993). Orchiectomy and nilutamide or placebo as treatment of metastatic prostatic cancer in a multinational double-blind randomized trial. *J. Urol.*, **149**, 77–83.

KELLY, W.K. & SCHER, H.I. (1993). Prostate specific antigen decline after antiandrogen withdrawal: the flutamide withdrawal syndrome. *J. Urol.*, **149**, 607–609.

LANDERS, J.P. & SPELSBERG, T.C. (1992). New concepts in steroid hormone action: transcription factors, proto-oncogenes, and the cascade model for steroid regulation of gene expression. *Crit. Rev. Eukaryot. Gene Expr.*, **2**, 19–63.

MARCONDES, J.A., MINNANI, S.L., LUTHOLD, W.W., WAJCHENBERG, B.L., SAMOJLIK, E. & KIRSCHNER, M.A. (1992). Treatment of hirsutism in women with flutamide. *Fertil. Steril.*, **57**, 543–547.

MOGUILEWSKY, M., FIET, J., TOURNEMINE, C. & RAYNAUD, J.P. (1986). Pharmacology of an antiandrogen, anandron, used as an adjuvant therapy in the treatment of prostate cancer. *J. Steroid Biochem.*, **24**, 139–146.

NAYFIELD, S.G., KARP, J.E., FORD, L.G., DORR, A. & KRAMER, B.S. (1991). Potential role of tamoxifen in prevention of breast cancer. *J. Natl Cancer Inst.*, **83**, 1450–1459.

POYET, P. & LABRIE, F. (1985). Comparison of the antiandrogenic/androgenic activities of flutamide, cyproterone acetate and megestrol acetate. *Mol. Cell. Endocrinol.*, **42**, 283–288.

SCHWALL, R., SCHMELZER, C.H., MATSUYAMA, E. & MASON, A.J. (1989). Multiple action of recombinant activin-A *in vivo*. *Endocrinology*, **125**, 1420–1423.

SOGANI, P.C., VAGAIWALA, M.R. & WHITMORE Jr, W.F. (1984). Experience with flutamide in patients with advanced prostatic cancer without prior endocrine therapy. *Cancer*, **54**, 744–750.

STEINSAPIR, J., MORA, G. & MULDOON, T.G. (1991). Effects of steroidal and non-steroidal antiandrogens on the androgen binding properties of the rat ventral prostate androgen receptor. *Biochim. Biophys. Acta*, **1094**, 103–112.

TYRREL, C.J. (1992). Casodex: a pure non-steroidal antiandrogen used as monotherapy in advanced prostate cancer. *Prostate*, **4** (Suppl.), 97–104.

WEBER, J.P., WALSH, P.C., PETERS, C.A. & SPIVAK, J.L. (1991). Effect of reversible androgen deprivation on hemoglobin and serum immunoreactive erythropoietin in men. *Am. J. Hematol.*, **36**, 190–194.